# Understanding factors influencing home pregnancy test use among women in western Kenya: A qualitative analysis

**DOI:** 10.3389/frph.2023.1092001

**Published:** 2023-04-06

**Authors:** Christina Mazumder, Annabel Dollah, Rosebel Ouda, Moses Okombo, Judith Nyakina, Monica L. Makia, Julia C. Dettinger, Laurén Gómez, Mary Marwa, Ben Ochieng, Felix Abuna, Claire Gwayi-Chore, Jillian Pintye, John Kinuthia, Grace John-Stewart, James Pfeiffer, Melissa L. Mugambi

**Affiliations:** ^1^ Department of Global Health, University of Washington, Seattle, WA, United States; ^2^ Department of Research and Programs, Kenyatta National Hospital, Nairobi, Kenya; ^3^ Safe Water and AIDS Project, Kisumu, Kenya; ^4^ Kenya Medical Research Institute, Kisumu, Kenya; ^5^ University of Nairobi, Nairobi, Kenya; ^6^ Department of Epidemiology, University of Washington, Seattle, WA, United States; ^7^ Department of Medicine, University of Washington, Seattle, WA, United States; ^8^ Department of Pediatrics, University of Washington, Seattle, WA, United States

**Keywords:** antenatal care, western Kenya, home pregnancy tests, COM-B model, qualitative analysis

## Abstract

**Background:**

There are limited data on home pregnancy test use among women in low-and-middle-income countries. A prior survey found that only 20% of women in western Kenya used a home pregnancy test to confirm their pregnancies before going to antenatal care. This qualitative study aims to understand why women do not use home pregnancy tests in early pregnancy.

**Methods:**

From April 2021 to July 2021, we interviewed women from four antenatal care clinics in Homa Bay and Siaya counties. We recruited women previously enrolled in the PrEP Implementation for Mothers in Antenatal care (PrIMA) study, a cluster-randomized trial that evaluated the best approaches to implementing PrEP in maternal and child health clinics in Western Kenya (NCT03070600). Interviews were conducted *via* phone, audio recorded, translated, and transcribed verbatim. We coded and analyzed the transcripts to capture factors influencing women's capability, opportunity, and motivation to use home pregnancy tests.

**Results:**

We conducted 48 semistructured interviews with women aged 21–42 years. Twenty-seven women did not use a home pregnancy test in their most recent pregnancy. Seventeen of these women reported not using a home pregnancy test before. Lack of knowledge, mistrust in the accuracy of tests, preferring to rely on signs and symptoms of pregnancy or get a test from the health facility, cost, and accessibility were key barriers to home pregnancy test use.

**Conclusion:**

Improving the uptake of home pregnancy testing during early pregnancy will require efforts to enhance community knowledge of test use and associated benefits and reduce cost burdens by making tests more affordable and accessible.

## Introduction

Over 90% of women in Kenya are estimated to receive antenatal care (ANC) from a skilled provider; however, as of 2014, only 20% sought antenatal care before the fourth month of pregnancy. Observational evidence suggests that women who use home pregnancy tests are more likely to present for antenatal care early (1, 2), which could lead to earlier identification of risk factors and health problems (e.g., hypertension, HIV, and other sexually transmitted infections) and prompt prevention and treatment during pregnancy, ultimately contributing to improved mother and child outcomes (3, 4). However, there is limited literature documenting home pregnancy test use among women in low-and-middle-income countries. For example, a 2019 survey found that 20% of women enrolling in ANC clinics in western Kenya used a home pregnancy test to confirm their pregnancies before presenting for ANC—this figure is much lower than in high-income settings where roughly 75% of women use a home pregnancy test (1, 5). Most women reported they did not use a home pregnancy test because they did not think it was necessary, did not have the money, or did not know about it (1). Women's knowledge and attitudes toward home pregnancy testing, motivations for use, and influences on ANC uptake in Kenya remain unclear.

The Capability, Opportunity, Motivation, Behaviour (COM-B) model is a helpful tool for understanding and changing health-related behavior. The model suggests that behavior results from the interaction between three factors: capability (psychosocial and physical), opportunity (physical and social), and motivation (automatic and reflective). The model posits that changing a person's behavior requires changing one or more of these factors (6). This qualitative study aims to understand why so few pregnant women buy and use home pregnancy tests by applying the COM-B model to capture factors influencing their capability, opportunity, and motivation to use pregnancy tests. By understanding the factors that influence home pregnancy test use, we can design strategies that are more likely to improve the uptake of pregnancy tests and, potentially, early antenatal care attendance.

## Methods

### Study setting and participants

This study recruited women who resided or had previously resided in Homa Bay and Siaya counties in western Kenya (7). As of 2019, Homa Bay and Siaya counties accounted for approximately 4.5% or roughly 2 million of Kenya's total population, with the majority residing in rural areas (8). The fertility rates in Homa Bay (3.6) and Siaya (3.5) are slightly higher than the national average of 3.4 children per woman in her lifetime (9). Approximately 11.2% of females over the age of 3 in these counties have never attended school, compared to 17.6% at the national level (10). Fifty-nine percent or 333 of the maternal and child health clinics are government-run (11). There are limited data on the number of pharmacies in both counties. As of 2023, we could identify 77 legally registered pharmacies in the Kenya Pharmacy and Poisons Board online facilities register. However, this number is probably higher as it does not account for those pharmacies that may not have updated their county location details in the register, for example. It is estimated that the number of unregistered pharmacies totals three times the number of registered pharmacies (12).

We recruited women previously enrolled in the PrEP Implementation for Mothers in Antenatal care (PrIMA) trial (NCT03070600). PrIMA was a cluster-randomized trial conducted between January 2018 and January 2021 that evaluated the best approaches to implementing PrEP in 20 maternal and child health clinics in Homa Bay and Siaya counties (7). The PrIMA study included government-run health facilities that served 30–50 new antenatal care clients per month in the smaller dispensaries, health centers, or subcounty hospitals and 100–200 new antenatal care clients per month in the larger subcounty and regional referral hospitals. Women were eligible to participate in the trial if they were pregnant, HIV negative, at least 15 years of age, and planned to reside in the study area for at least 1 year and receive postpartum and infant care services at the facility. We purposefully recruited women who had previously participated in the PrIMA trial, completed a baseline survey on pregnancy testing practices, and agreed to be recontacted after the trial. Separate approval for this study was obtained after the PrIMA trial. We stratified our sample based on home pregnancy test use and presentation for ANC in the first trimester of pregnancy. We selected 4 of the 20 health facilities to recruit the women—two in Homa Bay and two in Siaya counties. The four facilities were logistically accessible, served 50–200 new antenatal care clients per month, and provided a sufficient sample of women to meet the desired strata characteristics.

Interviewers screened participants for eligibility by reviewing a randomized list of 166 potentially eligible women (grouped by facility) who could be contacted *via* phone and were interested in participating in the study. Women were eligible to participate if they were between the ages of 15–44 years, HIV negative, and able and willing to provide informed consent. Altogether we enrolled 48 women, comprising the first 12 women from each facility who could be reached and were eligible, which was deemed sufficient to reach data saturation based on our experiences and prior studies (13, 14). Among potential participants contacted, no one declined to participate.

### Data collection

From April 2021 to July 2021, four qualitative interviewers (two women and two men) in western Kenya conducted individual interviews *via* phone. Interviewers were trained social scientists who had previously conducted qualitative work focused on community health, HIV prevention, and women's health. In response to the COVID-19 pandemic, the local ethics review committee established guidelines to minimize exposure to COVID-19 for study participants and staff. To comply with these guidelines, we conducted phone interviews instead of in-person visits whenever feasible. When in-person visits were necessary, study staff adhered to strict guidelines, including social distancing, masking, and room sanitization to reduce the risk of exposure. To ensure the validity and quality of the data collected, we conducted the interviews in a quiet and private location, and participants were encouraged to do the same. In addition, we used a structured interview protocol to standardize the interview process, and all interviews were audio recorded to verify the accuracy of the data collected. While interviewers typically had a reliable phone connection, there were a few instances where limited connectivity interrupted the interviews. However, the interviewers made every effort to address the connectivity issues and completed all interviews. Interviewers used an audio recording device to capture each study participant's voice in their comfortable language, primarily Dholuo, Swahili, or English. Interviews ranged from 17 to 64 min (*n* = 44).

One of the goals of the interview was to understand why women did not use home pregnancy tests to confirm their pregnancy. In addition, the interview guide included topics on care-seeking during early pregnancy, women's experiences using home pregnancy tests, HIV self-tests, and PrEP, and preferences for pharmacy-based delivery of these products and services. Before the interviews, study staff and interviewers familiar with the study context reviewed the guide to ensure that questions would be relevant and understandable to the study population. At the beginning of each interview, the interviewers obtained consent from all participants and conducted a brief survey covering participant demographics, pregnancy history, and experiences with community pharmacies. After the sessions concluded, interviewers prepared short debrief reports summarizing significant findings. Interviewers transcribed and translated all audio recordings into English as needed and stored the data in a secure, shared drive accessible only by study team members.

### Data analysis

In this study, we used a grounded theory approach, that is, we sought to explain why women do not use pregnancy tests based on participant accounts and experiences (15). We analyzed the data using multiple iterative steps guided by established qualitative analysis methods (16, 17). To organize and synthesize the data, we used manual coding methods and qualitative software tools, including Dedoose and ATLAS.ti, depending on access to and familiarity with the tools among the team members. The software tools provided a platform for us to upload our transcripts, code and categorize the data, and identify key themes as part of the analysis. Given that this study was part of a larger project understanding women's experiences with antenatal care, we developed an initial set of codes based on the interview guide and identified sections of the data in which women discussed their reasons for or against pregnancy test use. Six team members, including all interviewers, initially coded all transcripts independently and then coded additional transcripts during a second confirmatory round to assess for discrepancies. Next, one team member derived *in vivo* codes from women's responses indicating why they used or did not use pregnancy tests. Finally, three team members further refined and categorized the *in vivo* codes using the COM-B framework. Overall, data analysis was an iterative process consisting of immersing ourselves in the data by reading the debrief reports and transcripts, developing codes, coding transcripts, and identifying key themes and patterns across transcripts. Team meetings provided an opportunity to discuss the themes and potential discrepancies and establish consensus.

## Results

We conducted 48 semistructured interviews—43 *via* phone and 5 in person. Women's ages ranged from 21 to 42 years, with an average age of 29. The number of times women reported being pregnant ranged from 1 to 6, with a median of two pregnancies overall. Twenty-one women reported using a home pregnancy test, while 27 reported not using it in their most recent pregnancy. Of the women who reported not using a pregnancy test in their most recent pregnancy, 17 reported not having used a home pregnancy test before. Across sociodemographic characteristics, women who reported using a home pregnancy test in their most recent pregnancy were not notably different from women who did not use a home pregnancy test. However, the frequency of use of pharmacies in the past 12 months preceding the interview tended to be higher among users (Table 1). In describing our results, we pay particular attention to nonusers and highlight significant themes that capture why women did not use a home pregnancy test in early pregnancy, organized by the COM-B constructs of capability, opportunity, and motivation. The factors from the COM-B model that emerged as significant barriers to pregnancy testing included psychological capability, physical opportunity, and reflective motivation.

**Table 1:** Characteristics of users and nonusers of pregnancy tests.

Characteristic	Users (*N* = 21)	Nonusers (*N* = 27)
Age, median (IQR)	27 (26–30)	28 (24–34)
Current education status		
Primary school	1	5
Secondary/high school	7	8
University/college	8	7
Polytechnic	3	2
Not currently enrolled	2	5
Employment status		
Unemployed	11	6
Salaried	4	7
Regular hourly work	1	2
Irregularly hourly work	4	6
Other	1	6
Income earned per month, median (IQR)	5,000 (0–25,000)	5,000 (500–10,000)
Travel time to the nearest community pharmacy		
0–15 min	12	17
15–30 min	9	9
30 min—1 h	0	1
Over 1 h	0	0
Travel time to the nearest clinic		
0–15 min	7	7
15–30 min	12	13
30 min—1 h	2	6
Over 1 h	0	1
Number of pregnancies, median (IQR)	2 (1–3)^a^	3 (2–5)^b^
How often pharmacy was visited in the last 12 months		
Every month	2	0
Every 2 or 3 months	10	5
1 or 2 times a year	9	16
Never	0	6

^a^ Number of users = 20.

^b^ Number of nonusers = 24.

### Capability to use pregnancy tests

Psychological capability refers to whether women know about home pregnancy tests, when and how to use them, and where to access them. Seventeen women had not used a home pregnancy test before and, therefore, did not express confidence in their ability to use or, in some cases, access a home pregnancy test. For example, one woman stated: “I don’t know where they are bought or even how to use them … I cannot even think of using it … It is because I do not know how to use it. So, I will go to the hospital” (24-year-old woman). Uncertainty of pregnancy status was also a significant theme influencing one's capability to use a pregnancy test. Six women reported they felt sick and did not know they were pregnant until they went to the health facility for a check-up.

For example, one woman described how a friend encouraged her to seek medical care when she was unwell:

 “I started an antenatal clinic when I was four months pregnant … I did not know that I was pregnant. It was just a headache and vomiting … A friend of mine who lives nearby advised me. We are very close. So, when I explained to her those problems, she told me to go to the hospital. She told me that I could be pregnant. She gave me 100 shillings for transport. I presented to the hospital as a sick patient. I was tested for pregnancy when I explained the health problems which I had.” (26-year-old woman)

In another instance, a woman's husband encouraged her to seek care for her illness:

“Because by that time I used to fall very sick. I suffered from severe headache, I was vomiting a lot and I did not have any appetite. This went on for around two months and then my husband told me that we should both go to the hospital so as to find out what was happening to me. So I went and explained to the doctor how I was feeling and he told me that they wanted to first test for pregnancy because they had already tested for malaria and other diseases before and I was not suffering from any. They then tested me for pregnancy and it was positive.” (26-year-old woman)

### Opportunity to use pregnancy tests

Physical opportunity refers to whether women have the resources to obtain pregnancy tests or physically access locations that distribute them. For example, one woman did not have the money to buy pregnancy tests and suggested that it was more convenient to go to a hospital where testing services are offered for free rather than incurring expenses:

“Because I am a married woman, I just wanted to go to the hospital to be checked. I did not want to go to the chemist because of the expenses of using money to buy pregnancy test kit. Because I knew at the hospital the services were to be done for free … Aah, for me, if it is cheap, I can buy it. If it is affordable people can buy it at the chemist level.” (39-year-old woman)

One woman indicated that she was not close enough to a pharmacy and so could not get access to a pregnancy test: “The chemist is far from me if I had the chemist near me then maybe I would have just tested. That is why I went to the hospital to be tested” (22-year-old woman). Another woman said that if she had tests readily available at home, she would have used them: “I think the reason why I went to the hospital is because I didn’t have kits. Had I had one in the house I would have just done it in the house and see the outcome” (36-year-old woman).

Social opportunity refers to prevailing social norms or individual contacts that might expose women to perspectives and behaviors and influence their decision to use a pregnancy test. In some cases, women lacked social opportunities that would have otherwise encouraged them to use a pregnancy test or know how much it costs:

“I was not told to go and buy it. I was given a book when I reported to the doctor and went to where the pregnancy test was done. So, I was not told to buy it. I paid the fees of 300 shillings for the lab. That is what I paid … It is better to go to the hospital directly … because you can find that it is more expensive than the money you could use at the hospital. You can find the hospital only needs 300 shillings while the test kit is 500 shillings and you also do not have money. So, I do not see the need.” (38-year-old woman)

However, women who used pregnancy tests indicated they were affordable, with prices ranging from 30 KES (∼USD 0.30) to 50 KES (∼USD 0.50). For example, one woman said:

“Now, there is one reason why I went to the pharmacy. One will need 150 shillings so as to get the test but from the pharmacy the same test is being sold at 30 shillings. So, I feel that it is cheaper, and it saves you time and cost of going to the hospital for the test.” (27-year-old woman)

### Motivation to use pregnancy tests

Reflective motivation refers to the extent to which women value and prioritize pregnancy testing and the benefits it will bring and consider it necessary compared to other behaviors. Mistrust in the accuracy of pregnancy tests was a significant theme influencing women's motivation to use a pregnancy test, as captured by one woman:

“I did not test by myself; I knew that after I was tested in the lab then that was the truth. I did not bother to test by myself, I knew that if I test by myself then it would cheat me. I mean I cannot believe in it.” (36-year-old woman)

Women's negative perceptions of pharmacy-supplied products and pharmacy staff may have contributed to a lack of trust in purchasing pregnancy tests from the pharmacy, as reported by one woman: “I don’t trust the pregnancy kits at the pharmacy shelf … I think they give abnormal … they don’t give like the exact response. That is my thought, I am not sure. I have never tried” (24-year-old woman). Furthermore, women's awareness of, experiences with, and positive perceptions of health facilities meant confirming their pregnancy at the health facility was a more top-of-mind and desirable option.

“I went to the hospital to confirm the pregnancy … and later, I started the clinic at the same hospital. I chose (name of hospital) because it is a public facility which has experienced personnel … Yes I was tested at the hospital … No tangible reason but I saw it wise to go to the health facility.” (31-year-old woman)

One woman who had experienced a prior miscarriage was more cautious about ensuring she had no problems during pregnancy and, therefore, chose to confirm her pregnancy at a health facility:

“At first, I had lost my child so I had just to go for counseling and what because the pregnancy was … it just took some months then I had another pregnancy … you know sometimes when you are pregnant, you would like to know how your health is, if you are able to handle the pregnancy or if at all you have any problem. And if there is a problem then they get to treat you.” (34-year-old woman)

Some women may have missed a period or experienced other body changes that were signs of pregnancy and chose not to use a pregnancy test because confirming pregnancy based on signs and symptoms was sufficient, as reported by one woman:

“No, I did not use it but as months went by, I realized that changes were taking part in my body. Therefore, I knew that I was pregnant … I did not look for a pregnancy test kit because I knew that I was pregnant. So, there was no need.” (38-year-old woman)

However, other women who also experienced signs and symptoms of pregnancy chose to use a pregnancy on their own as it was quicker and easier compared to confirming at the health facility, which took time and effort.

“Antenatal care clinic, I think it is a place where you need to know after knowing that you are pregnant. So at least you just go there to confirm whether you are pregnant, but most of the time as mothers we go after knowing that we are pregnant. That is according to how the technology has also gone. So, you should know very well that it is sad just to line up, just to check whether you are pregnant or not. So, we at least go for the easiest by buying the pregnancy kit at around 30 bob (shillings).” (25-year-old woman)

“I knew that when I come here, I’ll be told to go to the lab so that I can be tested. That stress of being given that small bottle to go and get your urine so that you are tested is what I was trying to avoid. So, I had to do it myself first to confirm.” (34-year-old woman)

## Discussion

Urine home pregnancy tests were introduced to the global market in the 1970s and continue to provide women with an easy and discrete way to assess their pregnancy status (18). However, there are limited data on home pregnancy test use in western Kenya, and in one study, few women reported using home pregnancy tests in early pregnancy (1). This study aimed to understand why women who had previously attended antenatal care at public health facilities in Homa Bay and Siaya counties did not initially use home pregnancy tests to confirm their pregnancies. Women described multiple factors influencing their capability, opportunity, and motivation to use a home pregnancy test.

Some women did not know they could use a home pregnancy test to confirm their pregnancies, consistent with prior findings from a preliminary survey (1). These women may have had limited exposure to messages reinforcing home pregnancy test use through local media platforms, school education, friends, partners, or other community sensitization efforts. Few studies have explored the barriers to home pregnancy testing in Kenya and other countries in the broader eastern and southern African region. One study from South Africa also showed a lack of awareness of home pregnancy tests among women who did not use them (2). In addition, consistent with other studies, some women could not distinguish signs and symptoms of pregnancy, such as nausea, vomiting, or fatigue, from other common ailments in the region, such as malaria. In turn, sickness prompted women to visit the health facility for a check-up (19, 20).

Failure to afford or access home pregnancy tests hampered women's opportunity to use the tests. An assessment of the home pregnancy test market in Kenya suggests that tests are widely available in both private and public sectors, with prices ranging from USD 0.30 to USD 4 (21). Interestingly, public sector clinics might sometimes charge more for the tests than pharmacies, with prices ranging from USD 0.99 to USD 1.40 among public facilities visited. In our study, one user of a pregnancy test pointed out that the pharmacy charged approximately USD 0.30 for a test while the health facility charged USD 1.50; therefore, she selected the cheaper option. However, given the variability in price points among pharmacies, some prices may remain out of reach of the average woman from the study region. Although the WHO recommends making home pregnancy tests available outside of the health facility setting and studies have advocated for free pregnancy testing options, there have not been wide-scale efforts to ensure the availability of free or subsidized tests in western Kenya (22, 23).

The belief that tests were inaccurate or did not provide any value when women already knew they were pregnant meant that many women were not motivated to use pregnancy tests. Our findings are consistent with other studies that have reported a lack of trust in pregnancy test accuracy as a barrier to self-testing (5, 24). For example, a qualitative study in South Africa described trust concerns among young women who used pregnancy tests—specifically, upon repeated testing efforts; the women did not trust the results and felt the need to confirm their results at the clinic (24). A survey conducted in the US found that concerns about test accuracy were more pronounced in adolescents. The authors hypothesized that irregular menstrual periods or uncertainty in knowing the date of one's last menstrual period might interfere with pregnancy test result interpretation and compound the notion that the test results are inaccurate (5). In this study, mistrust in pharmacies and pharmacy staff might have exacerbated the belief that tests were inaccurate. In Kenya, the number of unregistered pharmacies far exceeds that of registered pharmacies, with 5,033 pharmacies estimated to be legally registered (25, 26). Additionally, the people who typically provide services at pharmacies, particularly smaller ones in rural areas, might not have a pharmacy degree or diploma (27). Therefore, the general community often perceives pharmacies to stock poor-quality or expired products and to be staffed by less well-trained individuals (28).

In this study, women reported prioritizing competing behaviors to confirm their pregnancy, including relying on pregnancy signs and symptoms or going directly to the health facility. Social and cultural norms surrounding pregnancy and childbirth mean that women are more likely to perceive pregnancy as a natural process in which they wait to recognize changes in their bodies and are sure of their pregnancy status before seeking antenatal care services (19). Additionally, there has been growing community sensitization around antenatal care-seeking, which might influence women's capability and opportunity to initially seek care at the health facility to confirm their pregnancy status (4). However, in certain circumstances, these approaches might delay the process of confirming pregnancy because women may not notice early signs of pregnancy or may mistake them for other conditions. Ultimately, the goal is to ensure that women gain timely access to beneficial preventive services during pregnancy. Therefore, multicomponent strategies influencing one's capability, opportunity, and motivation to conduct a pregnancy test and initiate antenatal care early are needed.

Several behavior change techniques might improve the uptake of pregnancy testing in early pregnancy (29, 30). Providing women with information about the benefits of early testing, such as making more informed decisions about their pregnancy and accessing early prenatal care through leaflets, posters, health provider recommendations, or public awareness campaigns, can improve women's capability and motivation to use pregnancy tests (31). We found that women who used pregnancy tests typically suspected they were pregnant based on their knowledge and awareness of pregnancy's physical signs and symptoms and wanted to confirm their suspicions before going to the antenatal care clinic. Additionally, making pregnancy tests more accessible, such as providing them for free or at low cost at pharmacies and other convenient locations, can encourage more women to get tested (32). One study from Madagascar showed that providing free pregnancy tests by community health workers at home improved the uptake of pregnancy testing and the number of women who knew they were pregnant and sought antenatal care services (33). Generally, women who used pregnancy tests in this study found them to be affordable and cheaper than going to the clinic to confirm their pregnancies. Therefore, efforts to educate women should also create awareness about pricing and where tests can be obtained. Finally, instilling confidence in women that pharmacies are a reliable source of healthcare will be paramount through investing in public education campaigns that explain the role of pharmacies in the healthcare system and increasing industry regulation (28). Given disparities in access to well-resourced pharmacies legally registered in rural areas, future research should explore the types of pharmacies women are likely to frequent, registered vs. unregistered, and whether this has any implication on their perception of trust in pharmacies.

Our study has some notable strengths. First, we used the COM-B model to systematically identify factors influencing women's capability, opportunity, and motivation to use a pregnancy test independently. The COM-B model sets the stage for developing theory-informed strategies to improve the uptake of pregnancy testing (29). Second, through individual interviews with 48 women, we obtained rich and detailed data about women's perspectives and experiences with pregnancy testing. We acknowledge that we conducted the interviews using mobile devices, which requires reliable coverage and data availability; a few instances occurred where we could not capture participant responses completely due to connectivity issues. We also acknowledge that our study population comprised women who attended government-run antenatal care clinics in Homa Bay and Siaya, were relatively older (the youngest participant was 21 years old), and were more likely to be in stable partnerships. Therefore, the perspectives shared might not necessarily represent the views of the broader population in the region and, in particular, younger, single women, who are likely to have different care-seeking behaviors and attitudes toward pharmacies and pregnancy testing (34, 35). Despite these limitations, this is one of the first studies to qualitatively explore barriers to pregnancy testing in the Kenyan region.

## Conclusions

There are little data on women's knowledge and attitude toward pregnancy tests in Kenya and the broader east and southern African region. Therefore, this study used the COM-B model to identify factors influencing pregnancy test use. Women described several reasons why they chose not to use pregnancy tests, including lack of knowledge, mistrust in the accuracy of tests, preferring to rely on signs and symptoms of pregnancy or obtain a test from the health facility, cost, and accessibility. While pregnancy is a unique experience in a woman's life, home pregnancy tests are a simple tool that can potentially protect the mother and child from adverse outcomes through early linkage to antenatal care services and preventive services. Recommendations to improve pregnancy testing during early pregnancy center on improving community knowledge on test use and associated benefits and reducing cost burdens by making test kits more affordable and accessible.

## Data Availability

The raw data supporting the conclusions of this article will be made available by the authors upon request, acknowledging that the data will be anonymized and that, in certain circumstances, we might need to obtain approval from ethical review committees to prevent any breaches of confidentiality.
